# Patient-reported Outcome Measures for Assessing Spectacle Independence after Implantation of Monofocal or Extended Depth of Focus (EDOF) Intraocular Lenses with Various Degrees of Monovision

**DOI:** 10.1055/a-2559-0878

**Published:** 2025-04-16

**Authors:** Katja Iselin, Claude Kaufmann, Martin K. Schmid, Michael Thiel, Frantisek Sanak, Kathrin Golla, Ying-Yu Melody Hedinger

**Affiliations:** Augenklinik, Luzerner Kantonsspital, Luzern, Switzerland

**Keywords:** spectacle independence, monovision, extended depth of focus EDOF, patient-reported outcome measures, PROMs, Brillenunabhängigkeit, Monovision, Extended Depth of Focus, EDOF, Patient-reported Outcome Measures, PROMs

## Abstract

**Purpose** Cataract surgery aiming for emmetropia in one eye and various degrees of myopia in the contralateral eye (monovision) is a popular strategy to improve spectacle independence. The aim of this study was to use patient-reported outcome measures to assess spectacle independence after implanting aspheric monofocal or extended depth of focus (EDOF) intraocular lenses (IOLs) with various degrees of monovision.

**Methods** All patients with bilateral cataract surgery between 2021 and 2024 aiming for micromonovision (− 0.5 to − 0.75 D), mini monovision (− 1.0 to − 1.5 D) or full monovision (− 1.75 to − 2.5 D) after cataract surgery with implantation of either aspheric monofocal IOLs (Tecnis ZCB00, Johnson & Johnson) or EDOF-IOLs (Tecnis Eyhance, Johnson & Johnson) were included in this study. Patients were implanted with either a monofocal IOL or an EDOF IOL in both eyes, and were aiming for emmetropia in the dominant eye. Six months postoperatively, all patients were contacted by telephone and asked to report their outcomes using a structured questionnaire investigating their spectacle usage for various daily activities and their overall satisfaction.

**Results** Thirty-two patients in the monofocal group and 88 patients in the EDOF group completed the questionnaire. In both groups, 22% of patients reported that they were entirely spectacle independent for all daily activities. However, the proportion of patients who used spectacles for at least 50% of the time was 41% in the monofocal group and 16% in the EDOF group. The percentages of patients who were able to perform computer work without spectacles with micro-, mini-, or full monovision were 27%, 67%, and 77% in the monofocal group and 61%, 60%, and 90% in the EDOF group, respectively. Spectacle-free reading of a smartphone or tablet was possible for 17%, 75%, and 71% of all patients with monofocal IOL and for 38%, 50%, and 90% with EDOF IOL, depending on the degree of monovision. Patient satisfaction was generally high with a maximum score of 4.9 out of possible 5.0 points in the full monovision EDOF group.

**Conclusion** Patients aiming for spectacle independence with monovision achieve better results when implanted with EDOF IOLs than with aspheric monofocal IOLs. Even with EDOF IOL, it is necessary to aim for full monovision (− 1.75 to − 2.5 D) in order to achieve spectacle independence for computer work.

## Zusammenfassung

**Hintergrund** Monovision mit Ziel Emmetropie am dominanten Auge und verschiedenen Graden an Myopie am kontralateralen Auge ist eine beliebte Strategie zum Erreichen einer Brillenunabhängigkeit nach Kataraktoperation. Ziel dieser Studie war es, anhand von Patient-reported Outcome Measures die Brillenunabhängigkeit nach Implantation von asphärischen monofokalen oder von Extended-Depth-of-Focus-Intraokularlinsen (EDOF-IOL) mit verschiedenen Graden an Monovision zu beurteilen.

**Methoden** In die Studie aufgenommen wurden alle Patienten, die sich in den Jahren 2020 und 2024 einer beidseitigen Kataraktoperation mit dem Ziel einer Monovision unterzogen. Den Patienten wurde beidseits entweder monofokale IOL (Tecnis ZCB00, Johnson & Johnson) oder EDOF-IOL (Tecnis Eyhance, Johnson & Johnson) implantiert, wobei entweder eine Mikromonovision (− 0,5 bis − 0,75 dpt), eine Minimonovision (− 1,0 bis − 1,50 dpt) oder eine Vollmonovision (− 1,75 bis − 2,5 dpt) angestrebt wurde. Sechs Monate nach der Operation wurden alle Patienten telefonisch kontaktiert und gebeten, ihre Einschätzung der Ergebnisse anhand eines strukturierten Fragebogens mitzuteilen, in dem die Brillennutzung für verschiedene tägliche Aktivitäten und die allgemeine Zufriedenheit abgefragt wurden.

**Ergebnisse** Die Einschätzungen konnten von 32 Patienten in der monofokalen Gruppe und 88 Patienten in der EDOF-Gruppe erhoben werden. In beiden Gruppen gaben 22% der Patienten an, bei allen täglichen Aktivitäten völlig brillenunabhängig zu sein. Allerdings betrug der Anteil der Patienten, die mindestens 50% der Zeit eine Brille trugen, 41% in der monofokalen Gruppe und 16% in der EDOF-Gruppe. Der Anteil der Patienten, die mit Mikro-, Mini- oder Vollmonovision ohne Brille am Computer arbeiten konnten, betrug 27%, 67% bzw. 77% in der monofokalen Gruppe und 61%, 60% bzw. 90% in der EDOF-Gruppe. Das brillenlose Lesen eines Smartphones oder Tablets war bei 17%, 75% bzw. 71% aller Patienten mit monofokalen IOL und bei 38%, 50% bzw. 90% mit EDOF-IOL möglich, je nach Grad der Monovision. Die Patientenzufriedenheit war im Allgemeinen hoch, mit einer maximalen Bewertung von 4,9 von 5,0 möglichen Punkten in der EDOF-Subgruppe mit Vollmonovision.

**Schlussfolgerung** Patienten, die eine Brillenunabhängigkeit mit Monovision anstreben, erzielen mit EDOF-IOLs bessere Ergebnisse als mit asphärischen monofokalen IOLs. Auch mit EDOF-IOL ist es notwendig, eine volle Monovision (− 1,75 bis − 2,5 dpt) anzustreben, um Brillenunabhängigkeit bei der Computerarbeit zu erreichen.

## Background

In recent years, cataract surgery has evolved from a simple replacement of the cloudy lens with a clear artificial lens to a refractive procedure. As a result, the expectations of patients undergoing cataract surgery have risen, with many now wishing to be “spectacle free” post-surgery. Spectacle independence can be achieved with trifocal lenses; however, these lenses are expensive and often have side effects such as light phenomena 1 .

Monovision is a less cost-intensive alternative for achieving spectacle independence with fewer optical light phenomena 2 . It is a method whereby the dominant eye is corrected for distance vision and the non-dominant eye is corrected for near to mid-range vision. The amount of intended residual myopia can also be adjusted according to the demands of the patient (i.e., micro-, mini- or full mono-vision).

To achieve monovision, either monofocal or extended-depth-of-focus (EDOF) intraocular lenses (IOLs) can be implanted. While monofocal IOLs have a single focal point, EDOF IOLs can slightly extend the range of vision by elongating the focal point to increase spectacle independence 3 .

The aim of this study was to use patient-reported outcomes (PROMs) to assess spectacle independence after implanting aspheric monofocal or EDOF IOLs with various degrees of monovision during cataract surgery.

## Patients and Methods

### Study patients

All patients who underwent bilateral cataract surgery between 2020 and 2024, aiming for monovision with either a EDOF or monofocal IOL in the non-dominant eye (− 0.5 D to − 2.5 D), and who completed the study questionnaire ≥ 6 months after surgery, were included in the study. Patients were excluded if they were aged ≤ 18 years, with vision-related ocular co-morbidities, such as macular pathologies, were unwilling or unable to report outcomes, or had no informed consent.

### Study design

This study was performed at the Department of Ophthalmology, Lucerne Cantonal Hospital, and approved by the ethics committee for northwest and central Switzerland (BASEC No. 2021-02525). All patients provided written consent for the collection, processing, and publication of their data.

Allocation of an individual patient to a cataract surgeon (n = 16) was based on standard in-house guidelines according to the availability of staff and surgical capacity and was not adjusted for the study. The refractive strategy was defined during the preliminary examination. Patients were implanted with either a monofocal (Tecnis1 ZCB00 or Tecnis Toric II, Johnson & Johnson) or EDOF IOL (Tecnis Eyhance or Tecnis Eyhance Toric II, Johnson & Johnson) in both eyes, aiming for emmetropia in the dominant eye. Patients were offered both lens types, however the EDOF lens was associated with additional costs for the patient, while the monofocal lens came without extra costs. In the non-dominant eye, the different monovision powers were defined as micro-monovision (− 0.5 to − 0.75 D), mini-monovision (− 1.0 to − 1.50 D) or full monovision (− 1.75 to − 2.5 D). The calculation of the intraocular lenses was based on optical biometry (IOL Master 700, Carl Zeiss Meditec AG) using the Barret Universal II formula for total keratometry and selecting the IOL power closest to the intended target. Corneal astigmatism exceeding 0.75 D was corrected using a toric lens design. Post-operative follow-up of patients was conducted by the referring ophthalmologists in private practice.

Six months post-surgery, patients were followed up by telephone and asked to complete a structured questionnaire that has been previously used in patients with standard aspheric IOLs 4 or refractive and diffractive EDOF IOLs (Supplemental Fig. 1) 5 . The questionnaire was completed in a 20-minute telephone interview with a paramedical staff member. The questionnaire asked patients about their post-operative use of spectacles in daily life and their general satisfaction with their choice of lens (measured via a questionnaire with scores ranging from 1 = not satisfied, to 5 = very satisfied). Patients who could not be contacted after two attempts and did not react to written invitation were excluded from the study.

### Statistical methods

Statistical analyses were performed using GraphPadPrism (Version 10.1.2) statistical software package. A p-value < 0.05 was considered statistically significant.

## Results

Overall, between 2020 and 2024, 547 patients underwent bilateral cataract surgery with monovision and 120 patients (22%) completed the study questionnaire at 6 months. Of these patients, 32 had monovision surgery with the monofocal IOL and 88 with the EDOF IOL ( Table 1 ). Mean (± SD) age of the patients was 75 (± 6.8) years for monofocal IOL and 74 (± 7.0) years for EDOF IOL (p = 0.8357). Approximately half of the patients in both groups were female (monofocal: 53%; EDOF: 57%, p = 0.9462).

**Table:** **Table 1** Spectacle dependence and patient satisfaction after implantation of monofocal or EDOF intraocular lenses.

	Monofocal n = 32	EDOF n = 88	p-value
Postoperative spectacles, n (%)			p ≥ 0.9999 ^b^
Yes	25 (78)	69 (78)	–
No	7 (22)	19 (22)	–
Type of postoperative spectacles, n (%) ^a^			
Reading spectacles, total	9 (28)	48 (55)	*p = 0.0131 ^b^
Prefabricated reading spectacles	5 (16)	30 (34)	p = 0.0684 ^b^
Near vision spectacles from the optician	4 (13)	18 (20)	p = 0.4275 ^b^
Distance spectacles	2 (6)	5 (6)	p > 0.9999 ^b^
Varifocal spectacles	15 (47)	22 (25)	*p = 0.0268 ^b^
Duration of spectacle wear, n (%)			*p = 0.0168 ^b^
Never	7 (22)	19 (22)	–
Only rarely for special tasks (< 20% of the time)	7 (22)	43 (49)	–
Occasionally (20 – 50% of the time)	5 (16)	12 (14)	–
Most of the time (> 50% of the time)	4 (13)	7 (8)	–
Always (100% of the time)	9 (28)	7 (8)	–
Activity without spectacles, n/n (%)			
Reading books/newspaper	9/32 (28)	24/88 (27)	p > 0.9999 ^b^
Reading with tablet/smartphone	15/30 (50)	40/87 (46)	p = 0.8324 ^b^
Computer work	15/27 (56)	52/81 (64)	p = 0.4943 ^b^
Household/garden	22/32 (69)	73/87 (84)	p = 0.0768 ^b^
Watching TV	19/32 (59)	75/86 (87)	**p = 0.0017 ^b^
Driving car	14/26 (54)	63/74 (85)	**p = 0.0023 ^b^
Satisfaction with the choice of lens, (score 1 – 5)			
Median/mean IOL satisfaction	5/4.65	5/4.68	p = 0.8684 ^c^
^a^ Patients could have more than one type of postoperative spectacles; ^b^ Fisherʼs exact test; ^c^ Mann-Whitney test; * significant; ** highly significant; EDOF: extended depth of focus; IOL: intraocular lens			

**Fig. 1:**
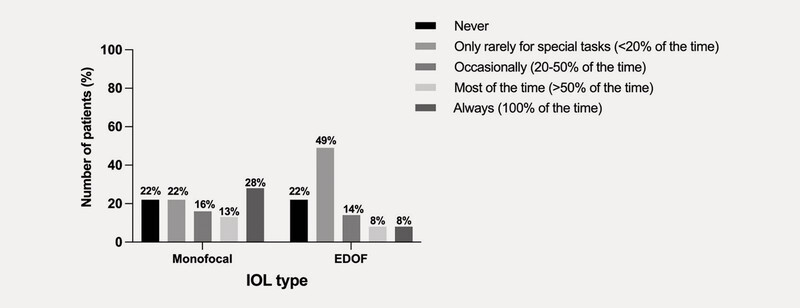
Overall spectacle dependency.

**Fig. 2:**
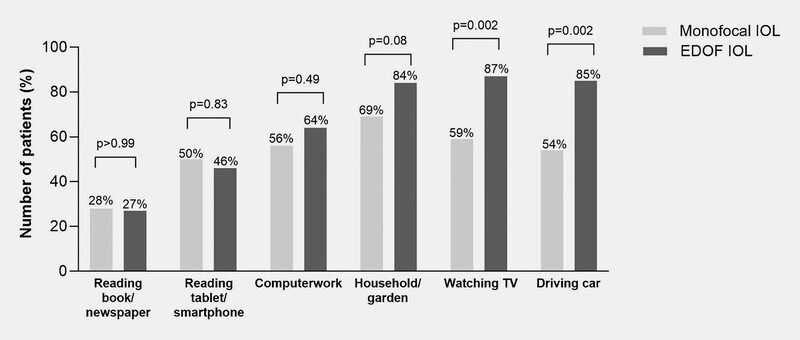
Spectacle use for daily activities.

With both types of IOL, 22% of patients reported being completely independent of spectacles ( Table 1 ; Fig. 1 ). Duration of spectacle wear was significantly less in the EDOF patients versus the monofocal patients (p = 0.02; Table 1 ). Overall, 13/32 (41%) patients with monofocal IOL and 14/88 (16%) patients with EDOF IOL reported wearing spectacles for > 50% of the time ( Table 1 ; Fig. 1 ). Use of distance spectacles was low in both groups (6%; Table 1 ). Significantly more patients in the monofocal group wore varifocal spectacles post-surgery than in the EDOF group (monofocal: 47% vs. EDOF: 25%; p = 0.03), whereas significantly more patients in the EDOF group wore simple monofocal reading spectacles post-surgery (monofocal: 28%; EDOF: 55%; p = 0.01).Regarding spectacle use for daily activities, significantly more patients in the EDOF group reported not needing spectacles when watching TV (87% vs. 59%; p = 0.002) and driving a car (85% vs. 54%; p = 0.002; Table 1 ; Fig. 2 ) versus those in the monofocal group. Spectacle-free reading of books/newspapers (monofocal: 28%; EDOF: 27%) and iPad/smartphone (monofocal: 50%; EDOF: 46%) was comparable between groups ( Table 1 ; Fig. 2 ). Patient satisfaction with lens choice was high, with a mean score of 4.7 in both groups.

**Fig. 3:**
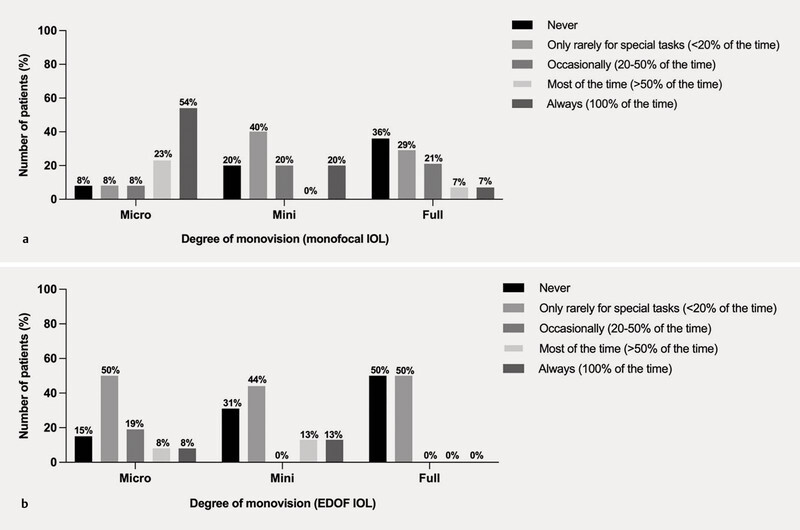
Duration of spectacle wear by degree of monovision with the monofocal IOL ( **a** ) and EDOF IOL ( **b** ).

**Fig. 4:**
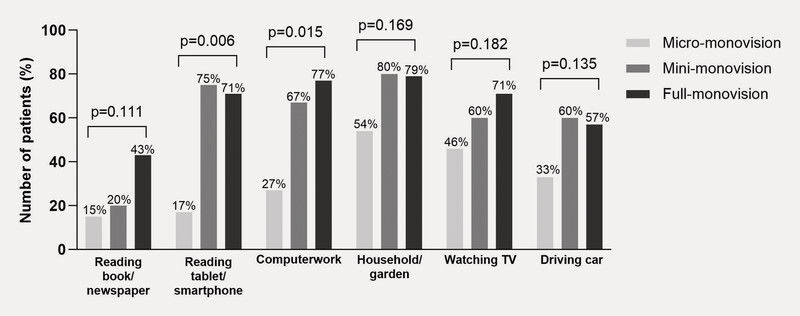
Spectacle independence during daily activities by degree of monovision with the monofocal IOL.

**Fig. 5:**
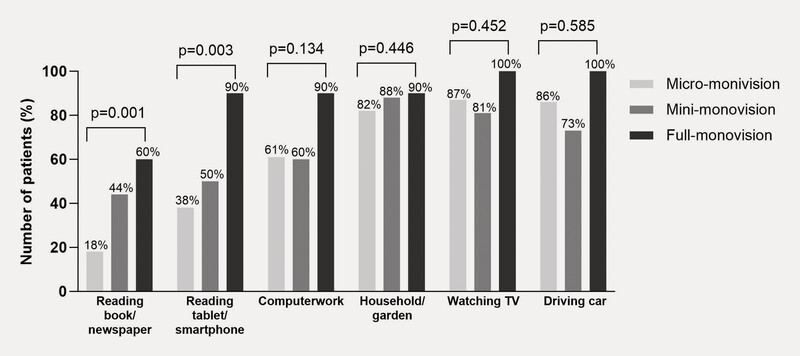
Spectacle independence during daily activities by degree of monovision with the EDOF IOL.

When analysing spectacle independence by degree of monovision (i.e., micro, mini-, and full-monovision), there was a trend for increased spectacle independence with increasing degree of monovision in the EDOF group (p = 0.006; Table 3 ; Fig. 3 b ), but not in the monofocal group (p = 0.08; Table 2 ; Fig. 3 a ). Spectacle-free reading of a tablet/smartphone was possible for 17%, 75%, and 71% of patients with monofocal IOL ( Table 2 ; Fig. 4 ) and for 38%, 50%, and 90% with EDOF IOL ( Table 3 ; Fig. 5 ), depending on the degree of monovision. Computer work without spectacles with micro-, mini-, or full-monovision was reported by 27%, 67%, and 77% of patients with monofocal IOL ( Table 2 ; Fig. 4 ) and 61%, 60%, and 90% of patients with EDOF IOL ( Table 3 ; Fig. 5 ), respectively. Statistical analyses showed evidence of a trend in spectacle independence with increasing degrees of monovision for tablet/smartphone reading with both lens types (monofocal: p = 0.006; EDOF: p = 0.003), for computer work with monofocal (p = 0.02), and for reading books/newspapers with EDOF (p = 0.001; Tables 2 and 3 , Figs. 4 and 5 ). Patient satisfaction was generally high with a maximum score of 4.9 in the full monovision EDOF group ( Tables 2 and 3 ).

**Table:** **Table 3** Patient demographics, spectacle dependence and patient satisfaction after implantation of EDOF intraocular lenses, by degree of monovision.

	Micro-Monovision n = 62	Mini-Monovision n = 16	Full-Monovision n = 10	p-value
Demographics				
Sex, female, n (%)	34 (55)	10 (63)	6 (60)	p = 0.8905 ^b^
Age, mean (SD), years	75.4 (6.925)	70.8 (6.4)	74.5 (6.8)	p = 0.0560 ^c,f^
Postoperative spectacles, n (%)				**p = 0.0063 ^d^
Yes	53 (85)	11 (69)	5 (50)	–
No	9 (15)	5 (31)	5 (50)	–
Type of postoperative spectacles, n (%) ^a^				
Reading spectacles, total	40 (65)	6 (38)	2 (20)	**p = 0.0026 ^d^
Prefabricated reading spectacles	25 (40)	3 (19)	2 (20)	p = 0.0834 ^d^
Near vision spectacles from the optician	15 (24)	3 (19)	0 (0)	p = 0.0922 ^d^
Distance spectacles	2 (3)	2 (13)	1 (10)	p = 0.1943 ^b^
Varifocal spectacles	15 (24)	5 (31)	2 (20)	p = 0.7370 ^b^
Duration of spectacle wear, n (%)				p = 0.0969 ^b^
Never	9 (15)	5 (31)	5 (50)	–
Only rarely for special tasks (< 20% of the time)	31 (50)	7 (44)	5 (50)	–
Occasionally (20 – 50% of the time)	12 (19)	0 (0)	0 (0)	–
Most of the time (> 50% of the time)	5 (8)	2 (13)	0 (0)	–
Always (100% of the time)	5 (8)	2 (13)	0 (0)	–
Activity without spectacles n/n (%)				–
Reading books/newspaper	11/62 (18)	7/16 (44)	6/10 (60)	**p = 0.0013 ^d^
Reading with tablet/smartphone	23/61 (38)	8/16 (50)	9/10 (90)	**p = 0.0031 ^d^
Computer work	34/56 (61)	9/15 (60)	9/10 (90)	p = 0.1345 ^d^
Household/garden	50/61 (82)	14/16 (88)	9/10 (90)	p = 0.4466 ^d^
Watching TV	52/60 (87)	13/16 (81)	10/10 (100)	p = 0.4527 ^d^
Driving car	42/49 (86)	11/15 (73)	10/10 (100)	p = 0.5856 ^d^
Satisfaction with the choice of lens (score 1 – 5)				
Median/mean IOL satisfaction	5/4.7	4.5/4.3	5/4.9	*p = 0.0308 ^e,g^
^a^ Patients could have more than one type of postoperative spectacles. ^b^ Fisherʼs exact test; ^c^ One-way ANOVA; ^d^ Cochran-Armitage test for trend; ^e^ Kruskal-Wallis test; ^f^ Post-hoc Tukeyʼs multiple comparisons test, adjusted p = 0.0436* for micro versus mini; ^g^ Dunnʼs multiple comparisons test, ns; * significant; ** highly significant; EDOF: extended depth of focus; IOL: intraocular lens; ns: not significant; SD: standard deviation				

**Table:** **Table 2** Patient demographics, spectacle dependence and patient satisfaction by degree of monovision, after implantation of monofocal intraocular lenses.

	Micro-Monovision n = 13	Mini-Monovision n = 5	Full-Monovision n = 14	p-value
Demographics				
Sex, female, n (%)	7 (54)	2 (40)	8 (57)	p = 0.8959 ^b^
Age, mean (SD), years	76.8 (6.6)	78.2 (3.7)	71.2 (6.6)	*p = 0.0387 ^c,f^
Postoperative spectacles, n (%)				p = 0.0782 ^d^
Yes	12 (92)	4 (80)	9 (64)	–
No	1 (8)	1 (20)	5 (36)	–
Type of postoperative spectacles, n (%) ^a^				
Reading spectacles, total	2 (15)	2 (40)	5 (36)	p = 0.4920 ^b^
Prefabricated reading spectacles	0	2 (40)	3 (21)	p = 0.0845 ^b^
Near vision spectacles from the optician	2 (15)	0	2 (14)	p > 0.9999 ^b^
Distance spectacles	0	0	2 (14)	p = 0.6331 ^b^
Varifocal spectacles	10 (77)	2 (40)	3 (21)	**p = 0.0040 ^d^
Duration of spectacle wear, n (%)				p = 0.0741 ^b^
Never	1 (8)	1 (20)	5 (36)	–
Only rarely for special tasks (< 20% of the time)	1 (8)	2 (40)	4 (29)	–
Occasionally (20 – 50% of the time)	1 (8)	1 (20)	3 (21)	–
Most of the time (> 50% of the time)	3 (23)	0 (0)	1 (7)	–
Always (100% of the time)	7 (54)	1 (20)	1 (7)	–
Activity without spectacles, n/n (%)				
Reading books/newspaper	2/13 (15)	1/5 (20)	6/14 (43)	p = 0.1112 ^d^
Reading with tablet/smartphone	2/12 (17)	3/4 (75)	10/14 (71)	**p = 0.0059 ^d^
Computer work	3/11 (27)	2/3 (67)	10/13 (77)	*p = 0.0152 ^d^
Household/garden	7/13 (54)	4/5 (80)	11/14 (79)	p = 0.1688 ^d^
Watching TV	6/13 (46)	3/5 (60)	10/14 (71)	p = 0.1817 ^d^
Driving car	3/9 (33)	3/5 (60)	8/12 (57)	p = 0.1352 ^d^
Satisfaction with the choice of lens, (score 1 – 5)				
Median/mean IOL satisfaction	5/4.7	5/4.4	5/4.7	p = 0.6696 ^e^
^a^ Patients could have more than one type of postoperative spectacles. ^b^ Fisherʼs exact test; ^c^ One-way ANOVA; ^d^ Cochran-Armitage test for trend; ^d^ Kruskal-Wallis test; ^e^ Post-hoc Tukeyʼs multiple comparisons test, ns; * significant; ** highly significant; IOL: intraocular lens; ns: not significant; SD: standard deviation				

## Discussion

This study assessed spectacle independence and patient satisfaction after implanting aspheric monofocal or EODF IOLs with various degrees of monovision during cataract surgery. The PROMs collected highlight important considerations when advising cataract patients: although complete spectacle independence with monovision is possible, this is by no means guaranteed, as shown by the fact that only 22% of both groups were completely spectacle-free. In evaluating spectacle use for daily activities of everyday life, we found that approximately half of patients in both groups were able to read on their smartphone without spectacles, while significantly more patients in the EDOF group were able to watch TV, or drive without spectacles.

Patients can also be informed that their choice of IOL type will most likely determine the type of spectacle lens they will use postoperatively if required, as significantly more patients used varifocals in the monofocal IOL group, whereas significantly more patients in the EDOF group used reading spectacles only. Patients can also be informed that a monovision solution with both monofocal and EDOF IOLs leads to consistently high satisfaction rates, as shown by an average score of approximately 4.6 out of a possible 5.0 points.

Broken down by degree of monovision, spectacle independence increased in both groups with increasing myopia of the non-dominant eye. As expected, however, the proportion of patients independent of spectacles with increasing myopia was higher in the EDOF group than in the monofocal group because EDOF lenses, unlike monofocal lenses, may support intermediate visual acuity by the overlapping ranges of extended depth of focus of the two lenses implanted.

Interestingly, the overall number of patients requiring spectacles of any kind post-implantation with EDOF IOLs was high in our study compared with findings in the published literature 6 , 7 . However, here we used PROMs, which have some advantages regarding data generalizability but are not as standardized as clinical measures and make study comparisons challenging 8 . In addition, definitions of ‘spectacle independence’ can vary greatly from study to study, further complicating inter-study comparisons 9 . Our findings support those of other previously published studies showing high spectacle independence for far vision and high patient satisfaction following monovision surgery 2 , 10 . Furthermore, they show that monovision can be used to achieve a relevant percentage of spectacle-free vision, suggesting that this solution be offered to patients who are not eligible for multifocality 2 .

This study has several strengths. First, the study was conducted in an unselected cohort of patients in a real-world setting of a hospital ophthalmology clinic, without preoperative contact lens testing of monovision as it is usually done in refractive patients with clear lens exchange. Second, the study answers clinically relevant questions based not on defocus curves and modular transfer functions but on the patientʼs reported visual experience. Technical parameters examined in clinical research only provide indirect indications of patientsʼ well-being in everyday life. In contrast, one of the main tasks of PROMs is to identify those procedures that bring the greatest benefit to patients. A third and important strength of this study is the long waiting period of 6 months between surgery and the interview. Allowing a longer time until follow up means that the patient has more time to adapt and “get used” to the new lenses, whereas studies obtaining PROMs earlier may collect feedback in a period where the patient has not completely adapted. This may also explain the somewhat lower proportion of patients who are spectacle free in our study compared with previous studies, as patients in our study had more time to evaluate their need for, and purchase, additional spectacles.

Limitations of the study include the small patient numbers who responded, particularly in the monofocal group. However, since postoperative care was provided by the referring ophthalmologists and the institution collecting the PROMs was not aware of the postoperative outcome, there was no bias in contacting patients depending on their results. The study does not allow to comment on any relation between preoperative refraction and the decision to choose monofocal IOLs or EDOF IOLs. The lack of postoperative refraction and the evaluation of the achieved rather than the intended refractive result is also a limiting factor, as a refractive error may have affected patientsʼ satisfaction. More patients with the EDOF lens reported driving post-surgery without spectacles; however, it is difficult to know whether these patients were driving without spectacles because their visual acuity was good enough or whether they were simply doing it regardless of their visual acuity. The questionnaire would have benefited from the addition of questions relating to contrast and nighttime vision as these are two important functions that may have provided further insight into the daily activities of patientsʼ post-surgery. Furthermore, the expectations and reported outcomes of patients receiving EDOF lenses may have been influenced by the additional expense associated with this type of lens.

In summary, patients aiming for spectacle independence with monovision achieve better results with the implantation of EDOF than with monofocal lenses, particularly for intermediate distances. However, even with EDOF IOLs, it is necessary to aim for full monovision (− 1.75 to − 2.5 D) in order to achieve independence from spectacles for computer work and reading on a smartphone. This recommendation differs from the usual manufacturer recommendations regarding the use of EDOF IOLs.

## Supporting Information

Supplemental Figure 1. Study Questionnaire Ergänzendes Material Supporting Information
