# Supplementary material for: Patient-reported Outcome Measures for Assessing Spectacle Independence after Implantation of Monofocal or Extended Depth of Focus (EDOF) Intraocular Lenses with Various Degrees of Monovision
Source: Klin Monbl Augenheilkd. 2025 Apr 16;242(4):363–71. doi: 10.1055/a-2559-0878 (PMC12020672; doi:10.1055/a-2559-0878)
Supplement: Supplementary file 1 — Ergänzendes Material [file 10-1055-a-2559-0878_kl0436-sup_0436_figure_1_questionnaire.pdf]

Supplemental Figure 1. Study Questionnaire

Datum: ____________________

Patientenetikette

Operateur/In: ______________ **Optiker/In:** _________________

OD: OP-Datum: _____________ **sphärische Zielrefraktion:**______________ ☐ **reduz. visuspotential**
OS: OP-Datum: _____________ **sphärische Zielrefraktion:**______________ ☐ **reduz. visuspotential**

☐ **mit Femtosekundenlaser** **Grund:**_____________

Linsentyp: **OD:** **OS:**

**Tec 1 sphärisch**	☐	☐	
**Torisch**	☐	☐	
**Eyhance sphärisch (EDOF)**		☐	☐
**Eyhance torisch (EDOF)**		☐	☐
**Lara sphärisch (EDOF)**		☐	☐
**Lara torisch (EDOF)**	☐	☐	
**Trifokal**	☐	☐	
**Trifokal torisch**	☐	☐	

Fragebogen nach der Operation des grauen Stars (6 Monate nach der Operation)

Datum: ________________ ☐ Telefon ☐ bei Kontrolluntersuch

1. **Tragen Sie jetzt, nach der Operation, eine Brille?**

☐ Ja
☐ Nein (brillenlos)

2. **Wenn Sie seit der Operation eine Brille tragen, um was für eine Brille handelt es sich?**

☐ nur Fertig-Lesebrille. Kosten: _______________
☐ Gleitsichtbrille für Ferne und Nähe. Kosten: _______________
☐ gleiche Gleitsichtbrille wie prä-OP
☐ Brille nur für die Ferne. Kosten: _______________
☐ gleiche Fernbrille wie prä-OP
☐ Brille nur für die Nähe. Kosten: _______________
☐ gleiche Nahbrille wie prä-OP
☐ sonstige Brille: _______________________

Neues Brillenrezept (sofern vorhanden): ☐ Optiker☐ pAA ☐ LUKS

	Sphäre	Cylinder	Achse	Add	Visus
**Rechtes Auge**					
**Linkes Auge**					

3. Wie gross ist der Anteil der Zeit, in der Sie aktuell eine Brille tragen?

☐ nie
☐ nur selten bei besonderen Aufgaben (weniger als 20% der Zeit)
☐ gelegentlich bei besonderen Aufgaben (20- 50% der Zeit)
☐ meistens (mehr als 50% der Zeit)
☐ immer

4. Wenn Sie in der vergangenen Woche eine Brille verwendet haben, für welche Tätigkeit?

ja:	nein:	Tätigkeit wird nicht ausgeführt:
☐	☐	☐ Autofahren tagsüber
☐	☐	☐ Autofahren nachts
☐	☐	☐ Einkaufen
☐	☐	☐ TV schauen
☐	☐	☐ Computer
☐	☐	☐ Haushalt/ Garten
☐	☐	☐ Lesen mit Tablet, Smartphone
☐	☐	☐ Lesen von Zeitungen + Büchern

☐ andere Tätigkeit: ____

5. Treten seit der Linsenimplantation unangenehme Lichtphämone beim Sehen (Halos) auf?

☐ Nein
☐ Ja:
☐ Sind Sie durch die Halos beim Autofahren gestört?
☐ Haben Sie deshalb das Fahren eingeschränkt?
☐ Haben Sie deshalb das Fahren aufgegeben?

6. Sind Sie mit der getroffenen Linsenwahl zufrieden?

☐ sehr zufrieden: ★★★★★
☐ zufrieden: ★★★★
☐ einigermassen zufrieden: ★★★
☐ eher unzufrieden: ★★
☐ sehr unzufrieden: ★

7. Wie gut fühlten Sie sich bei der Linsenwahl von uns beraten?

☐ sehr gut: ★★★★★
☐ gut: ★★★★
☐ eher gut: ★★★
☐ nicht so gut: ★★

☐ gar nicht gut: ★·

8. Sonstiges/Bemerkungen:
